# Parental Qualities, Family Dynamics, and Youth Mental Health: Navigating Interventions and Developmental Trajectories

**DOI:** 10.3390/children13060814

**Published:** 2026-06-12

**Authors:** Yosi Yaffe, Batel Hazan-Liran

**Affiliations:** Department of Special Education, Tel Hai Academic College, Kiryat Shmona 12208, Israel

The landscape of child and adolescent mental health has become increasingly complex, moving away from solely child-focused approaches to more holistic, systemic models that consider youth development within the context of the family environment [1]. This shift is particularly significant for families raising children with special needs, developmental delays, and psychological disabilities, as they face unique chronic stressors and structural barriers [2]. Contemporary clinical and developmental research highlights that the emotional and behavioral outcomes of these vulnerable populations are closely linked to parenting styles and parental well-being [3].

There have been ongoing gaps in understanding the specific mechanisms by which parental factors affect youth dealing with neurodevelopmental, psychiatric, and physical challenges [4,5]. Additionally, more clarity is needed on how structured interventions operate in real-world community settings [6]. This Special Issue aims to address these important issues by integrating advanced statistical modeling, qualitative insights, and clinical intervention data, with a particular focus on parenting children with special needs.

This collection of papers explores the specific parental qualities and psychological mechanisms that influence the behavioral and emotional development of children with clinical conditions. One study by Ankori et al., focusing on attention-deficit/hyperactivity disorder (ADHD) in Israeli families, shows that parental rejection and lack of warmth serve as a significant mediator. This mediator fully accounts for the relationships among parental anxious attachment and children’s behavioral problems. In another study, Yaffe et al. examined the impact of parenting practices in the context of autism spectrum disorder (ASD). Their path analysis revealed that authoritarian and permissive parenting styles directly increase child anxiety. In contrast, authoritative and permissive styles indirectly influence both youth anxiety and physical activity through the specific mechanism of parental encouragement of physical activity.

Further contextualizing the clinical needs of youth entering psychiatric care, Levkovich et al. explored age and gender variations in a large outpatient sample from a public hospital. They identified emotional dysregulation as a core transdiagnostic mechanism linking depressive symptoms to both internalizing and externalizing problems, particularly among older adolescent girls.

The Special Issue goes beyond examining micro-level family dynamics to investigate how parental stress and health literacy interact with healthcare systems, institutional frameworks, and early community interventions. Park and Ryu expanded the Theory of Planned Behavior and used structural modeling to show that parenting stress significantly reduces caregivers’ intentions to enroll children with disabilities in health promotion programs. This decline occurs primarily due to the erosion of subjective norms and perceived behavioral control. This finding highlights the importance of providing structural support and fostering self-efficacy, in addition to offering basic health education.

Lin conducted research on Taiwanese families with children who have developmental delays. She found that overcoming barriers to leisure in urban areas relies significantly on both personal and organizational health literacy. Resilient families view community leisure activities not just as recreational opportunities but also as structured tools for development that support readiness for school. Additionally, to address the stressful period before the diagnosis of early childhood neurodevelopmental delays, Masuda et al. assessed the effectiveness of the Group Positive Parenting Program for mothers of toddlers. Their study provided strong evidence that this scalable public health intervention can significantly reduce dysfunctional parenting practices, alleviate maternal depression, and help restore parenting confidence.

The contributions in this Special Issue provide profound insights into the family ecosystem of children with special needs. By mapping these intricate pathways, this body of work points toward vital future research directions: prioritizing longitudinal designs to track family dynamics across developmental transitions, multi-informant approaches to reduce reporting bias, and evaluation of digital, culturally tailored interventions. Ultimately, these studies offer invaluable clinical and community frameworks designed to foster caregiver resilience, enhance family well-being, and protect youth mental health globally.
